# Child and adolescent psychiatry training curriculum: a global trainee's perspective

**DOI:** 10.1192/bji.2020.8

**Published:** 2020-08

**Authors:** Sundar Gnanavel, Pawan Sharma, Antonin Sebela, Teresa Gomez Alemany, Jane Pei-Chen Chang, Mauro Victor de Medeiros Filho, Kwabena Kusi-Mensah, Fransiska Kaligis, Hidekazu Kato, Huu Kim Le, Kana Morimoto, Dmytro Martsenkovskyi, Darpan Kaur Mohinder Singh, Aditya Pawar, Asilay Seker, Napat Sittanomai, Sifat-e Syed, Utkarsh Karki, Arpit Parmar, Marcus Tan

**Affiliations:** 1 MD, MRCPsych, Higher Trainee, Child and Adolescent Mental Health Services, Tees, Esk and Wear NHS Foundation Trust, UK. Email: sundar221103@yahoo.com; 2 MD, Lecturer, Department of Psychiatry, Patan Academy of Health Sciences, Nepal; 3 MD, Postgraduate student, Department of Psychiatry, National Institute of Mental Health, Klecany, Czech Republic; 4 Psychiatry Trainee, Department of Psychiatry, Benito Menni Hospital, Barcelona, Spain; 5 MD, Consultant Psychiatrist, Department of Psychiatry, China Medical University Hospital, Taichung, Taiwan; 6 MD, Medical Assistant, Department of Psychiatry, University of Sao Paulo, Brazil; 7 MSc, Lead Clinician, Department of Psychiatry, Konfo Anokye Teaching Hospital, Kumasi, Ghana; 8 SpKJ(K), Lecturer, Division of Child and Adolescent Psychiatry, Faculty of Medicine, Universitas Indonesia, Jakarta, Indonesia; 9 MD, Graduate student, Department of Psychiatry, Nagoya University Graduate School of Medicine, Japan; 10 FRANZCP, Consultant Child and Adolescent Psychiatrist, Women's and Children's Health Network, Adelaide, Australia; 11 MD, Psychiatrist, Department of Psychiatry, Kyoto City Child Welfare Centre, Japan; 12 MD, Medical Doctor, Department of Psychiatry, Bogomolets National Medical University, Kiev, Ukraine; 13 DNB, Consultant, Satguru Mind Care Centre, Mumbai, India; 14 MD, Psychiatry Resident, Department of Psychiatry, Drexel University School of Medicine, Philadelphia, USA; 15 Child and Adolescent Psychiatry Trainee, Department of Psychiatry, Erciyes University Hospital, Turkey; 16 MD, Lecturer, Division of Child and Adolescent Psychiatry, Mahidol University, Bangkok, Thailand; 17 MD, Assistant Professor, Department of Psychiatry, Bangabandhu Sheikh Mujib Medical University, Dhaka, Bangladesh; 18 Senior Resident, Department of Child and Adolescent Psychiatry, National Institute of Mental Health and Neuro Sciences, Bengaluru, India; 19 Senior Resident, Department of Psychiatry, All India Institute of Medical Sciences, New Delhi, India; 20 ST5 Higher Trainee, South London and Maudsley NHS Foundation Trust, London, UK

**Keywords:** Child psychiatry, education and training, curriculum development

## Abstract

This article is a summary of perspectives on training curricula from child and adolescent psychiatry trainees globally. We aimed to identify the relative strengths, weaknesses and gaps in learning needs from a trainee's perspective. The 20 early-career child psychiatrists who contributed are from 16 countries and represent all the five continents. We could identify some global challenges as well as local/regional challenges that need to be addressed to develop competent child psychiatrists.

In 2015, a meta-analysis estimated the worldwide pooled prevalence of child and adolescent mental health disorders at 13.4%.^1^ It is estimated that approximately half of the population with mental health disorders experience illness onset at or before 14 years of age.^2^

The number of trained child and adolescent mental health professionals has been extremely low, particularly in low- and middle-income countries.^3^ A number of countries do not have specialised child and adolescent mental health training programmes. Instead, general psychiatrists and paediatricians try to partially fill in the void. It is also a well-recognised fact that across those countries that offer training in child psychiatry, the content and breadth of the curriculum is extremely variable and inconsistent.^4^

This article is a summary of perspectives from child and adolescent psychiatry (CAP) trainees across the globe regarding their respective training curricula. The contributors include 17 young child and adolescent psychiatrists from 16 countries currently working on the five continents: Asia: Bangladesh, Japan, India, Indonesia, Nepal, Taiwan and Thailand; Africa: Ghana; Oceania: Australia; Europe: the Czech Republic, Spain, Ukraine, the UK and Turkey; America: Brazil and the USA.

All of the 17 young psychiatrists are active in their respective national psychiatric organisations and have been commended for their contribution to the field as young psychiatrists. Ten of them were recipients of a Donald J. Cohen Fellowship Award from the International Association for Child and Adolescent Psychiatry and Allied Professions (IACAPAP); seven were recipients of a Fellowship Award from the Japanese Society for Psychiatry and Neurology (JSPN); four were recipients of a Fellowship Award from the World Psychiatric Association (WPA); and two were recipients of a Fellowship Award from the Royal Australia and New Zealand College of Psychiatry (RANZCP). One of the authors is chair of the CAP working group of the European Federation of Psychiatric Trainees (EFPT).

## Method

This study was planned as an outcome of a research promotion programme titled ‘Strategic Meeting for International Collaborative Research Development’, hosted by the Japanese Society of Psychiatry and Neurology in 2018. A semi-structured questionnaire was circulated among the above contributors focusing on the following four themes: (a) structure of their child psychiatry training curricula; (b) strengths of their respective curricula; (c) weaknesses in their curricula; (d) unmet training needs, suggestions/scope for improvement and novel developments in respective countries. Where possible, the first author counter-checked the information provided online and on the official websites of respective national organisations.

## Results

### Structure of training curricula

#### No separate CAP training programme

Some of the countries listed, such as Nepal, India, Ukraine and Spain, do not have a specialised CAP training programme. Instead, a 3- to 6-month rotation in CAP is inbuilt in the general psychiatry training. In India, some institutions offer a 1-year fellowship in CAP and a 3-year subspecialisation in CAP after general psychiatry training. Similarly, a 3- to 5-month fellowship in CAP is offered in some institutes in Ukraine. The UK, Australia, Japan, Indonesia and a recent programme in Bangladesh offer a 2- to 4-year training in CAP following general psychiatry training, which stands in addition to approximately 6 months’ training in CAP as part of general psychiatry training. Taiwan and Brazil offer a 1-year training in CAP following general psychiatry training.

#### CAP training following general psychiatry or paediatric training

Some countries, such as Turkey, have a separate CAP training programme. It is interesting to note that Thailand, the USA, Japan and the Czech Republic offer multiple pathways to specialise as a child psychiatrist after either general psychiatry or paediatric training. An integrated pathway also exists in the USA combining paediatric, general psychiatry and child psychiatry training.

#### Level of standardisation and differential pathways

In the USA and Japan clinical rotations can vary depending on the chosen programme. The UK seems to have a highly structured and homogeneous CAP curriculum across the country, as does Australia, albeit shorter in duration than the UK's (24 versus 36 months). In contrast, multiple pathways leading to CAP certification are available in Thailand.

### Relative strengths and weaknesses of individual curricula

The trainees identified a variety of strengths and weaknesses in their respective curricula. Some of the most common features are presented in this section.

#### Breadth and depth of curriculum

The comprehensiveness of the curriculum, covering all areas of CAP through a number of structured clinical rotations in which residents can explore distinct psychopathology, biopsychosocial formulations and evidence-based treatment for different diagnoses, was the most important aspect of curricula consistently mentioned by respondents. In total, 10/17 (59%) trainees felt that the breadth/depth of their curricula was comprehensive enough to enable them to practise CAP competently. The remainder cited lack of exposure to psychotherapeutic skills (systemic approach), forensic/medicolegal aspects, neurodevelopmental disorders and transition to adult facilities as major limitations of their curricula. Unsurprisingly, trainees with access to specialised CAP programmes tended to feel that their curriculum was more comprehensive compared with those whose CAP curricula were built into general psychiatry training.

#### Workload

Although it is desirable that trainees take a lead in the management of a number of patients in order to provide them with valuable experience in clinical settings, too large a case-load might be detrimental to the quality of training. According to our respondents, a very large case-load is the norm for trainees in low- and middle-income nations (e.g. Indonesia, India, Bangladesh, Ghana and Brazil) compared with the high-income nations (e.g. the UK, Australia). This could also be a reflection of the paucity of trained staff in child and adolescent mental health services in low- and middle-income countries.

#### Supervision by trainers

Regular protected hours dedicated to supervision was a common theme that trainees rated of high importance. Only 7/17 (41%) respondents mentioned receiving structured regular supervision. Trainees emphasised that regular supervision would strengthen their scientific criticism, incentivise the formulation of new questions and promote evidence-based literature search for further discussion. Lack of adequate supervision time in low- and middle-income countries was felt to be a consequence of the high demands made of the trained staff.

#### Training in psychotherapy

Mandatory supervised psychotherapy sessions and training were widely recognised as another important curricular feature. However, they were also cited as a common pitfall in most training programmes except those from Australia, the USA and UK. Lack of trained supervisors and time constraints imply extremely limited exposure for trainees to psychotherapy techniques. Note that, although training in psychotherapy theory is provided in some countries (e.g. India, Bangladesh), hands-on experience may not be mandatory. In fact, such sessions are mandatory in only a minority of the countries (5/16, 31%).

#### Academic training

It was encouraging to note provision of a rigorous academic teaching programme comprising case discussions, journal clubs and lectures wherever a CAP training programme exists. However, in those countries where CAP is essentially a part of general psychiatry training (e.g. Spain, India), the time allotted for CAP academic programmes is highly variable and inconsistent.

#### Research and networking

Research opportunities and networking are inbuilt in a number of CAP training programmes, particularly in high-income countries. More than half of respondents (12/17, 71%) said they were able to access adequate opportunities to participate in ongoing research projects. Some contributors stated that they could access these opportunities as part of general psychiatry training, although in some countries (e.g. Ukraine) these opportunities are scarce.

#### Bespoke opportunities

Programme flexibility in terms of bespoke opportunities tailored to individuals’ special/specific interests appears limited except in Japan, the USA, UK, Australia and Taiwan. Particularly, exposure to forensic CAP as a subspecialty seems to be extremely limited in most of the low- and middle-income nations.

#### Community services and school liaison

A general absence of training in community CAP services, school consultations and community services such as social work services was widely pointed out, except in the USA, Spain, UK and Australia.

#### Training in advocacy and leadership skills

Training in advocacy and leadership skills is beneficial to any professional, but it is especially important for CAP because of the essential requirement for liaison with schools and families and also because part of the job is about supporting the mental well-being of children and adolescents in greater institutions. However, it is an intended learning outcome in only few countries, such as the UK.

#### Examinations and workplace-based assessments

The sitting and passing of an exit examination (comprising theory and clinical case examinations) is required before the acquirement of certification. However, in the UK and Australia, great emphasis is placed on periodic workplace-based assessments (WPBAs) by clinical supervisors.

#### International collaborations

There are a number of initiatives with joint collaboration between clinicians working in high-income countries with those working in resource-stretched settings. There is an ongoing PhD programme in CAP at a tertiary care centre in Nepal (Kanti Hospital) in collaboration with the University of Oslo, Norway. The CAP training programme in Bangladesh welcomes input from child psychiatrists from Canada and Germany, including guest lectures. Bilateral collaboration between the University of Michigan and the Ghana College of Physicians as well as Universitas Indonesia's collaboration with the University of Hawaii are some other relevant examples.

## Discussion

### Limitations

It is acknowledged that the above summary is based on the perspectives of one or two young CAP psychiatrists from each of the countries represented and hence merits a careful interpretation. However, the contributors are in training or have trained in a variety of institutions/settings (not limited to major tertiary care hospitals). Also, the respondents were requested to provide their general perspective regarding CAP training in their respective countries rather than just commenting on their individual training programme.

### Implications

The study identified a number of common global challenges and some unique regional challenges in CAP training. It is reasonably clear that there is inadequate standardisation in CAP training. This lack of consistency and uniformity can impair creation of CAP professionals with the required knowledge, skills, expertise and competencies. It is encouraging to note the efforts of the European Union of Medical Specialists Section on Child and Adolescent Psychiatry (UEMS-CAP) and the European Federation of Psychiatric Trainees (EFPT) working to create a common European standard in CAP training.^5,6^ Similar regional efforts could be replicated elsewhere, for example Southeast Asia, South Asia and Latin America, for better homogenisation and devising a robust CAP training curriculum. The involvement of CAP trainees as the primary stake holders in this process is imperative. There is still a long way to go until global needs in CAP training are fulfilled.
